# The Inflamed Brain in Schizophrenia: The Convergence of Genetic and Environmental Risk Factors That Lead to Uncontrolled Neuroinflammation

**DOI:** 10.3389/fncel.2020.00274

**Published:** 2020-08-27

**Authors:** Ashley L. Comer, Micaël Carrier, Marie-Ève Tremblay, Alberto Cruz-Martín

**Affiliations:** ^1^Graduate Program for Neuroscience, Boston University, Boston, MA, United States; ^2^Department of Biology, Boston University, Boston, MA, United States; ^3^Neurophotonics Center, Boston University, Boston, MA, United States; ^4^Center for Systems Neuroscience, Boston University, Boston, MA, United States; ^5^Axe Neurosciences, Centre de Recherche du CHU de Québec, Université Laval, Québec City, QC, Canada; ^6^Division of Medical Sciences, University of Victoria, Victoria, BC, Canada; ^7^Department of Biochemistry and Molecular Biology, The University of British Columbia, Vancouver, BC, Canada; ^8^Department of Pharmacology and Experimental Therapeutics, Boston University, Boston, MA, United States

**Keywords:** neuroinflammation, microglia, schizophrenia, genes, Environment, risk factors, brain development, neurodevelopmental

## Abstract

Schizophrenia is a disorder with a heterogeneous etiology involving complex interplay between genetic and environmental risk factors. The immune system is now known to play vital roles in nervous system function and pathology through regulating neuronal and glial development, synaptic plasticity, and behavior. In this regard, the immune system is positioned as a common link between the seemingly diverse genetic and environmental risk factors for schizophrenia. Synthesizing information about how the immune-brain axis is affected by multiple factors and how these factors might interact in schizophrenia is necessary to better understand the pathogenesis of this disease. Such knowledge will aid in the development of more translatable animal models that may lead to effective therapeutic interventions. Here, we provide an overview of the genetic risk factors for schizophrenia that modulate immune function. We also explore environmental factors for schizophrenia including exposure to pollution, gut dysbiosis, maternal immune activation and early-life stress, and how the consequences of these risk factors are linked to microglial function and dysfunction. We also propose that morphological and signaling deficits of the blood-brain barrier, as observed in some individuals with schizophrenia, can act as a gateway between peripheral and central nervous system inflammation, thus affecting microglia in their essential functions. Finally, we describe the diverse roles that microglia play in response to neuroinflammation and their impact on brain development and homeostasis, as well as schizophrenia pathophysiology.

## Introduction

Schizophrenia (SCZ) is a prevalent mental illness without satisfactory treatment options. Approximately 20 million people worldwide are afflicted by this chronic and debilitating mental disorder (American Psychiatric Association, 2013; Whiteford et al., 2013). SCZ is characterized by a broad range of clinical manifestations including hallucinations, social and cognitive impairments, as well as disordered thinking and behavior that impair daily functioning (American Psychiatric Association, 2013). Current treatment options do not improve cognitive or negative symptoms, both of which contribute more significantly to the long-term prognosis of SCZ than positive symptoms (Lieberman et al., 2005; Green, 2006). More effective therapies for SCZ have lagged due to a lack of understanding of its underlying mechanisms.

Genome-wide association studies (GWAS) have identified novel susceptibility loci that confer greater risk for SCZ (Ripke et al., 2013; Li et al., 2017). These breakthroughs have enabled the characterization of genes that may shed light on the pathophysiology of SCZ. In addition, much progress has been made in preclinical studies focusing on environmental risk factors for SCZ and other neurodevelopmental disorders (NDD) that alter brain development such as psychosocial stress, maternal immune activation (MIA), and exposure to pollution (Bergdolt and Dunaevsky, 2019; Gomes et al., 2019; Horsdal et al., 2019). Although there are a multitude of genetic and environmental factors conferring increased risk for SCZ, recent work suggests that these factors converge by altering immune processes, which are known to play an essential role in shaping brain development (Müller and Schwarz, 2010; Stephan et al., 2012; Kroken et al., 2018). Indeed, elevated immune function and chemokine responses are found in SCZ and therapeutics that target immune function have shown some success in symptom reduction (Sommer et al., 2014; Frydecka et al., 2018; Kroken et al., 2018). Importantly, subclinical inflammation correlates with cognitive deficits in SCZ (Misiak et al., 2018), which are a critical determinant for the long-term prognosis of this disease. It is unclear how immune molecules regulate synaptic wiring during normal brain development and contribute to synaptic pathology in neuropsychiatric disorders. Causal links between specific immune molecules and altered synaptic connectivity within circuits implicated in neuropsychiatric disorders are currently lacking (Elmer and McAllister, 2012).

Microglia are central nervous system (CNS) phagocytes that, among their other roles, orchestrate innate immunity in the brain. Microglia have well-described roles in rapidly responding to inflammatory insults through dynamic surveillance of the CNS parenchyma (Nimmerjahn et al., 2005; Liu Y. U. et al., 2019) and clearing debris and apoptotic cells through phagocytosis (Ayata et al., 2018; Galloway et al., 2019). Recent studies have begun to uncover the diversity of microglia, which can have significantly different gene expression patterns across brain regions, in health and in pathological states, and at different developmental time points (Tay et al., 2017a; Hammond et al., 2019; Sankowski et al., 2019; Tan et al., 2020). These complex cells contribute to normal brain development and function by supporting the neuronal circuitry through synapse addition, elimination, maintenance, and plasticity (Hammond et al., 2018; Bohlen et al., 2019). Despite variability in the findings of several studies, there is evidence of microglial dysfunction in SCZ (Bayer et al., 1999; Hercher et al., 2014; Bloomfield et al., 2016; Trépanier et al., 2016; De Picker et al., 2017; Sellgren et al., 2019; Uranova et al., 2020). A key element to understand the pathogenesis of SCZ is to discern how genetic and environmental risk factors intersect to alter microglial function given. Furthermore, outstanding questions that remain to be answered are at what stage(s) of disease progression microglial function ameliorates or contributes to the pathology of SCZ, and what are the particular subtypes or phenotypes of microglia that could be targeted for therapeutic intervention.

In this review, we discuss the genetic and environmental risk factors for SCZ and how they converge to alter microglial function in response to systemic and central inflammation. Additionally, we highlight how these risk factors alter the indispensable functions of microglia during development, adolescence and adulthood. Limitations of the current knowledge are also addressed, and key future experiments are proposed. Understanding how the heterogeneous genetic and environmental risk factors for SCZ interact to reach a disease threshold and determine its progression is necessary for the development of more effective therapeutics.

## Genetic Risk Factors That Interplay With Immunological Responses

Schizophrenia is driven by genetic factors, as the risk for developing this disorder increases from 1% in the general populationto 50% in individuals with a diagnosed twin (Cardno and Gottesman, 2000; Stefansson et al., 2009). Recent ground-breaking genome-wide association studies (GWAS) have made progress in discovering loci throughout the genome that are associated with SCZ (Schizophrenia Psychiatric Genome-Wide Association Study Consortium, 2011; Ripke et al., 2013; Schizophrenia Working Group of the Psychiatric Genomics Consortium, 2014; Li et al., 2017; Dennison et al., 2019). These studies reveal that SCZ has a heterogeneous etiology, with genes likely conferring risk across the entire genome. This heterogeneity, in combination with environmental factors, has made it difficult to pinpoint which genes contribute to the disease pathology. Although the genetic determinants for SCZ are not well understood, evidence suggests that immune dysfunction and inflammation contribute to its pathophysiology (Trépanier et al., 2016; van Kesteren et al., 2017).

The major histocompatibility (MHC) locus is located on chromosome 6 and has the highest association to SCZ compared to any other loci across the genome (Shi et al., 2009; Stefansson et al., 2009; Schizophrenia Working Group of the Psychiatric Genomics Consortium, 2014). This region encodes genes that are involved in innate immunity. For instance, complement component 4A (*C4A*), located in the MHC locus, is highly associated with SCZ: specific structural variants and regulatory regions that increase the expression of *C4A* confer a greater risk for SCZ (Sekar et al., 2016). The complement cascade is part of the innate immune system that recognizes foreign pathogens and apoptotic cells, and tags them for destruction, such as through phagocytosis by macrophages (Veerhuis et al., 2011). Besides their established role in immune defense, complement proteins play a role in various stages of brain development including neurogenesis, cellular migration and synaptic development (Veerhuis et al., 2011; Lee et al., 2019). Ground-breaking work in the last 5-10 years have linked complement proteins to microglia-mediated pruning of synapses, suggesting that *C4A* could directly contribute to SCZ pathology (Stevens et al., 2007; Schafer et al., 2012; Hong et al., 2016).

In line with this, it was recently shown that increased expression of the mouse homologue of *C4A, C4b*, in medial prefrontal cortex (mPFC) layer (L) 2/3 pyramidal neurons led to a marked reduction in connectivity and decreased sociability in juvenile and adult mice, both of which mirrored the deficits seen in SCZ (Comer et al., 2020). These results suggest that *C4A* might contribute directly to pathology in SCZ. Although, the molecular mechanisms that link increased *C4* expression to synaptic loss remain unclear, overexpressing this neuroimmune gene led to increased localization of the postsynaptic protein PSD-95 to microglial lysosomes, suggesting upregulated microglia-dependent synaptic engulfment (Comer et al., 2020). Additionally, variation in *C4* structural alleles increases risk for autoimmune diseases and indicate that sex-differences in the *C4* gene might explain greater vulnerability to SCZ in males (Kamitaki et al., 2020). In another study, C4 serum levels were assessed at baseline and in a 1-year follow-up in a cohort of twenty-five patients with first episode psychosis that were taking either olanzapine or risperidone (Mondelli et al., 2020). Compared with responders to antipsychotic medication, non-responders showed significantly higher baseline C4 levels, suggesting that baseline expression of this immune gene can predict clinical outcome (Mondelli et al., 2020). Since this study focused on a limited number of markers, it is not clear however how psychosis progression correlates with levels of other immune genes. Lastly, the gene ‘CUB and sushi multiple domains 1‘ (*CSMD1*) is an important regulator of C4 that is expressed during early postnatal development (Kraus et al., 2006). Genetic variants located in the *CSMD1* and *CSMD2* genes have been linked to SCZ (Håvik et al., 2011) and their dysregulation led to deficits in general cognitive ability and executive function (Athanasiu et al., 2017), both of which are affected in SCZ. Conversely, a recent study showed that CSMD1 levels in the blood are decreased in SCZ, while antipsychotic treatment resulted in up-regulation of CSMD1 and improved cognitive symptoms (Liu Y. et al., 2019).

Transcriptomic and genomic studies have implicated alterations in key cytokines with SCZ, including increases in interferon regulatory factor 3 (*IRF3*) (Li et al., 2015), which is a major transcription factor in viral infection, and interferon gamma (*IFN*-γ), an important regulator of viral propagation (Paul-Samojedny et al., 2011). In support of neuroimmune genes altered in SCZ, other studies have found changes in pro-inflammatory interleukin 1 (*IL)-1*α (Katila et al., 1999), *IL-1*β (Katila et al., 1999; Sasayama et al., 2011), *IL-6* (Kalmady et al., 2014; Frydecka et al., 2015) and anti-inflammatory *IL-10* [reviewed in Gao et al. (2014)]. Several studies also investigated circulating C-reactive protein (CRP), IL-6, IL-1β, TNF-β, and TGF-β, which are also elevated at the mRNA level in people with SCZ, to determine their reliability as peripheral biomarkers (Kroken et al., 2018). However, other studies reported limited immune gene enrichment in SCZ (Pouget et al., 2016), highlighting the genetic complexity of the disease, in addition to possible variability between cohorts and confounding factors such as medication, among other challenges with GWAS.

Several GWAS have revealed that multiple immune receptors are associated with SCZ including the MHC receptors and Toll-like receptors (TLRs) (Purcell et al., 2009; Shi et al., 2009; Stefansson et al., 2009; Schizophrenia Psychiatric Genome-Wide Association Study Consortium, 2011; Schizophrenia Working Group of the Psychiatric Genomics Consortium, 2014). TLRs play a role in the recognition of microbe-derived molecular signals by innate immune cells including microglia [reviewed in Wright et al. (2001) and Lehner (2012)]. In addition to their established role in innate immunity, TLRs regulate early brain development (Mallard, 2012; Chen et al., 2019) via their effects on synaptic plasticity and neurogenesis (Barak et al., 2014). Other groups have shown alterations in TLR2 (Kang et al., 2013) and TLR4 (García-Bueno et al., 2016; MacDowell et al., 2017) in either the blood or post-mortem brain tissue of people with SCZ. Overall, these data have linked MHC signaling and other immune receptors pathway with the pathology of SCZ, However, the molecular underpinnings of their contribution to SCZ are not yet clear. It also still not understood how disruption in particular immune pathways contributes to specific cellular and behavioral hallmarks of this disorder, such as decreased gray matter volume.

To identify robust peripheral biomarkers that can predict SCZ pathology, researchers have compiled an architecture of genes observed in patients from multiple GWAS. A subset of overlapping genes from these studies identified candidates including *CD14, CLU, DPP4, EGR1, HSPD1, MHC* and *C4* genes (Pouget et al., 2016). Despite the identification of these candidate biomarkers, other studies highlight that the current literature does not provide sufficient evidence that increased inflammation is a hallmark of all SCZ cases (Kroken et al., 2018). Some studies have identified markers that are related to antigen presentation and immune activity (Pouget et al., 2016), whereas others have revealed changes in inflammatory cytokines (Hudson and Miller, 2018; Kroken et al., 2018). These studies together indicate that some cases or stages of SCZ may involve the innate and/or adaptive immune system. However, genetics only explains part of the susceptibility and pathophysiology of SCZ, which provides further support that environmental risk factors are also required to trigger the disease in most cases (Knuesel et al., 2014).

Lastly, SCZ-associated genes with diverse functions in the brain have also been implicated in inflammation (Brandon et al., 2009). For example, the gene Disrupted-in-Schizophrenia 1 (*DISC1*) was first found in a Scottish family with SCZ (St Clair et al., 1990) and subsequently in other populations worldwide (Chubb et al., 2008). Interestingly, the disruption of DISC1 protein in mice led to dysregulation of an immune-related network of genes that are perturbed in SCZ (Trossbach et al., 2019), suggesting that non-immune genes can modulate the expression of inflammatory gene networks. In support of this, in a dual-hit genetic-environmental mouse model of SCZ, where *DISC1* mutation was combined with MIA, transient administration of minocycline, an anti-inflammatory antibiotic drug, rescued electrophysiological and structural deficits during early postnatal development, as well as cognitive abilities in juvenile mice (Chini et al., 2020). It is clear that the expression of hundreds of genes is altered in SCZ, although it remains to be determined how the interaction between immune and non-immune pathways is implicated in this disorder. Overall, growing evidence suggests that immune gene dysfunction and inflammation both contribute to the pathophysiology of SCZ (Trépanier et al., 2016; van Kesteren et al., 2017).

## Exposure to Pollution Causes Neuroinflammation

**FIGURE 1:**
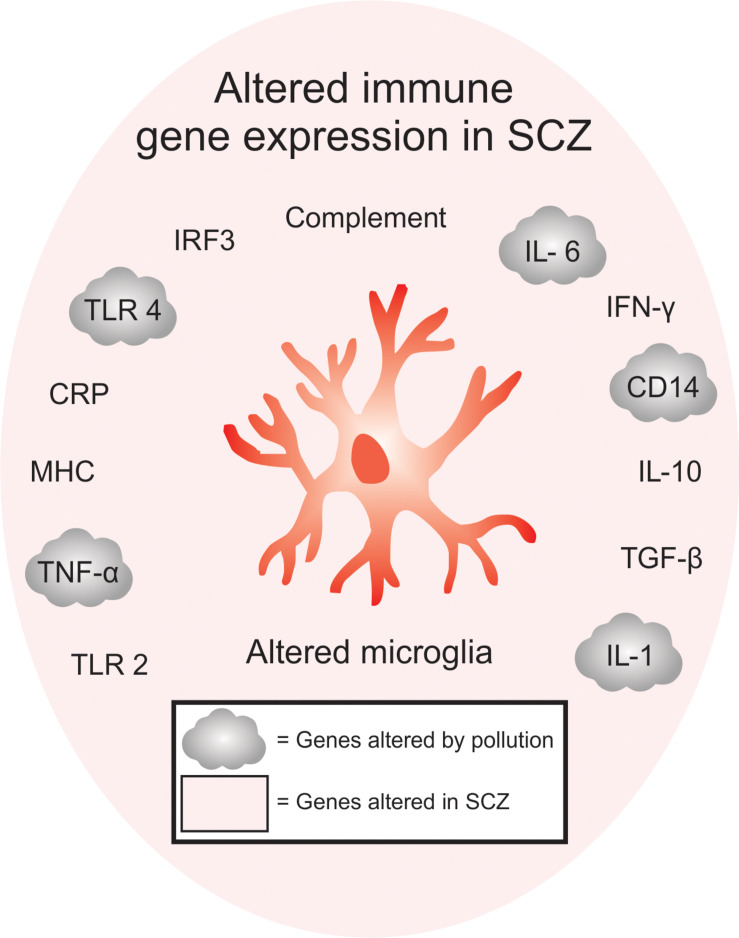
Overlap between immune-signaling genes that are associated with pollution and SCZ. Of the immune genes that are associated with SCZ, many are also found to be altered either in humans or animals exposed to pollutants, offering a genetic point of convergence between changes in pollution-mediated inflammatory signaling and SCZ. Inflammatory gene expression, including TLR2, TNF-α, MHC, CRP, TLR4, IRF3, complement pathway, IL-6, IFN-y, CD14, IL-10, TGF-β are altered in SCZ, while TNF-α, TLR4, IL-6, CD14 and IL-1 are altered in SCZ and after exposure to air pollution. These genes are specifically enriched in microglia.

The environment is becoming increasingly polluted from multiple sources. Traffic-related air pollution (TRAP), such as diesel exhaust (Inoue et al., 2006; Hartz et al., 2008; Block and Calderón-Garcidueñas, 2009; Bolton et al., 2017), is the result of the combustion of fossil fuels and can be modeled in the lab using elemental carbon (Newman et al., 2013) or by taking the finest particles (<200 nm) from TRAP and re-aerosolizing them into nanoparticulate matter (nPM). nPM is the most toxic component of TRAP, in terms of its impact on the brain (Davis et al., 2013). By-products of TRAP, such as ozone (O_3_), which can be generated from nitrogen oxide, can also be changed photochemically after their release from motor vehicles (Mumaw et al., 2016). Altogether, multiple paradigms are currently used in animal models to study the effects of air pollution on brain development (Davis et al., 2013; Newman et al., 2013; Woodward et al., 2017; Table 1). This work is particularly relevant when considering the epidemiological studies that link air pollution to SCZ pathogenesis (Horsdal et al., 2019). Indeed, many of the genes altered in SCZ overlap with genes that are affected by exposure to air pollution (Figure 1). Interestingly, immune genes, including those expressed by microglia, are at the center of this interaction (Peters et al., 2006; Genc et al., 2012).

**TABLE 1:** Overview of the effects of different pollutants on neuroinflammation.

Pollutant Expourse	Species	Age	Sample measurements	Phenotype	Articles
NO2 and PM	humans	8 years old (exposure in infancy)	serum levels	increase in IL-6 and IL-10 2017	Gruzieva et al., 2017
CO, NOx, NO2, and benzene	humans	longitudinal- from childhood to adulthood	SCZ diagnosis	increased risk of developing SCZ (only for exourse to benzene and CO	Pedersen et al., 2004
NOx, NO2 and PM	humans	longitudinal- from childhood to adulthood	Presense of psychosis	increased odds of psychotic experiences (only for exposure to NO2 and NOx)	Newbury et al., 2019
PM (2.5 um)	humans	young adults	plasma levelsapoptosis	apoptosis of endothelial cells, increased levels of circulating monocytes and T-cells, increased proinflammatory cytokines (IL-6, IL-1β, MCP-1, and MIP-1)	Pope et al., 2016
DEP	mice	embryonic (E18) to young adulthood (P30) (prenatalexposure)	cytokine ELISAs and IHC from hippocampus and parietal cortex	increased cytokines and altered morphology of microglia in male mice dependent on TLR4 signaling; altered cortical volume; increased microglia-neuron interactions in males	Bolton et al., 2012, 2017
DEP	mice	adults	olfactory bulb and hippocampus protein levels	increased lipid peroxidation and pro-inflammatory cytokines (IL-1α, IL-1β, IL-3, IL-6, and TNF-α). Increased expression of Iba1 and TSPO	Cole et al., 2016
DEP	mice	adult (prenatal exposure)	behavioral, brain protein and mRNA levels from PCF, HPC, hypothalamus and parietal cortexi	increased anxiety behaviors; Increased IL-1β and TLR4 in males and decreased IL-10	Bolton et al., 2013
nanoscale PM (<0.2 um)	mice	adults	neonatal cortical neurons	impaired neurnal differentiation; increased depressive behaviors	Davis et al., 2013
nanoscale PM (<0.2 um)	mice	adults	corpus callosum protein levels	increased complement protein deposition (C5, C5a and CD88) in brain but not serum; altered microglial morphology	Babadjouni et al., 2018
PM	mice	juvenile mice (postnatal day 11-15) (prenatal exposure)	cerebellum myelin density, cerebellum iron levels, RNAseq of cerebellum	increased inflammation signaling; increased iron inclusions; myelin sheath damage	Klocke et al., 2018
PM (2.5 um)	mice	adults (exposure in utero)	western blot, ELISA and IHC in temporal cortex	deficits in spatial memory; increase in COX2 and Arg1 protein, increase in GFAP reactivity, decreased cytokines levels in temporal cortex (IL-1α, IL-2, IL-4 IL-6, IL-10, IFN-γ, GM-CSF and TNF-α) and spleen (IL-2,IL-6, IL-10 and TNF-α)	Kulas et al., 2018
ultrafine elemental carbon	mice	adults (neonatal exposure)	behavioral assays, protein expression in the corpus callosum and ventricles	no changes observed in locomotion, learning, memory, impulsivity or anxiety behaviors, no changes in GFAP or MBP	Morris-Schaffer et al., 2019
ultrafine PM	mice	juvenile and early adulthood	hippocampus and amygdala transcript and protein, corpus callosum IHC, behavioral measures	lateral ventricle dilation; changes in cytokines, neurotransmitters and microglia activation markers in sex-dependent manner, hypomyelination, elevated glutamate, increased repetitive and impulsive behaviors	Allen et al., 2014, 2017
nanoscale PM (<0.2 um)	rats	gestation to adulthood	behavioral, protein levels in the adult hippocampus	70% decrease in adult hippocampal neurogenesis, 35% increase in Iba1 in the dentate gyrus; 75% decrease in tight junction protein of the BBB; impaired contextual memory, food-seeking and depressive-like behaviors	Woodward et al., 2018
nanoscale PM (<0.2 um)	rats/mice	*in vitro*: postnatal day 3, *in vivo*: adult	glial transcript measurements in culture (rat mized glial cultures) and hippocampus (mice)	TLR4-mediated increase in 2000 transcripts related to neuroinflammation and stress	Woodward et al., 2017
carbon black and DEP	rats/mice	adults	cultured microglia (mice BV-2 cells) and hippocampus (rat) protein levels	increased IL-6, TNF-a, and Iba1, increased caspase-3 mediated autophagy in microglia	Bai et al., 2019
ozone (O3), mixed vehicle exhaust	rats/mice	young and aged adults	serum levels	increased microglial proinflammatory response especially pronounced in aging mice	Mumaw et al., 2016

*PM: particulate matter, SCZ: schizophrenia, ELISA: enzyme-linked immunosorbent assay, IHC: immunohistochemistry, PFC: prefrontal cortex, HPC: hippocampus, mRNA: messenger RNA, IL: interleukin, TSPO: translocator protein, Iba: Ionized calcium binding adaptor molecule 1, TLR**:** toll-like receptor, TNFα: tumor necrosis factor alpha, MCP-1: monocyte chemoattractant protein-1, MIP-1: macrophage inflammatory protein 1, GFAP: glial fibrillary acidic protein, CD88: cluster of differentiation 88, COX-2: cyclooxygenase-2, IFNγ: interferon gamma, gm-csf: granulocyte-macrophage colony-stimulating factor, C: complement cascade, BBB: blood brain barrier.*

While the mechanisms involved in SCZ pathogenesis are still unclear, exposure to air pollution has been found to increase the expression of multiple inflammatory genes in humans and mouse models. Children exposed to TRAP have elevated circulating levels of pro-inflammatory cytokines, including IL-6, IL-1ß, CD14, and TNF-α, compared to children living in less-polluted cities (Calderón-Garcidueñas et al., 2008, 2015; Gruzieva et al., 2017). Additionally, nPM from air pollution induced a similar inflammatory cytokine signature in the circulation of healthy young adults, characterized by an elevation of IL-6, together with an increased density of inflammatory cells and microparticles, suggesting the occurrence of endothelial injury (Pope et al., 2016). In line with this, TRAP exposure in rodents increased IL-1α, IL-6 and TLR4 expression in the brain (Bos et al., 2012; Bolton et al., 2017). Pollution exposure especially impacted microglial TLR4 signaling in multiple mouse models involving TRAP (Woodward et al., 2017, 2018), O_3_ (Mumaw et al., 2016), or diesel exhaust particle (Bolton et al., 2017; Bai et al., 2019) exposure, by upregulating TLR4 in a MyD88-dependent pathway (Woodward et al., 2017). Male offspring were especially susceptible to these deleterious effects, showing greater changes in microglial TLR4 signaling that were accompanied by behavioral deficits in anxiety-like behavior, contextual and auditory cue fear conditioning and the forced swim test (Bolton et al., 2012, 2013; Bolton et al., 2017). Prolonged exposure to inflammatory molecules, such as IL-6, additionally led to neuroadaptive effects such as altered synaptic plasticity (Gruol, 2015). Therefore, exposure to pollution could alter brain development and function by causing increased expression of pro-inflammatory markers, a feature on which MIA models of SCZ rely on (Girgis et al., 2014).

TRAP alters brain development and increases the risk for SCZ (Pedersen et al., 2004; Woodward et al., 2015), however it is unclear if pollution-mediated changes in brain development or inflammatory signaling directly contribute to pathology. Recent work has studied the effects of chronic nPM exposure using a double-hit model where cortical neuronal cultures from exposed mice were re-exposed in culture. A double exposure to nPM reduced neurite outgrowth (Davis et al., 2013) while resulting in an inflammatory transcriptomic profile (Solaimani et al., 2017) in neuronal cultures. Another group showed that TRAP can reduce hippocampal neurogenesis by 70% in rats, which correlated with behavior deficits in object recognition, food-seeking behavior, and in the forced swim test (Woodward et al., 2018). These phenotypes were reproduced in mice using elemental carbon exposure (Morris-Schaffer et al., 2019). In the mouse brain, nPM exposure induced neuroinflammation evident through a microglia-mediated increase in TNF-α (Cheng et al., 2016). Furthermore, exposure to nPM led to altered microglia morphology and elevated levels of C5, C5a, and CD68 proteins, indicative of increased phagocytic activity, in the corpus callosum (Babadjouni et al., 2018) a region that is particularly reduced in volume in SCZ patients (Kubicki et al., 2005). Other work has highlighted the neurotoxicity of ultrafine particles (UFP), which induced pro-inflammatory signaling and lead to a long-lasting reduction of corpus callosum volume (Allen et al., 2017). Overall, neuroinflammation induced by pollution appears to have a substantial impact on the brain by altering axonal myelination (Cole et al., 2016). However, it is still unclear to what extent pollution-driven inflammation, compared to other risk factors, drives myelination deficits in SCZ. Taken together, the inflammatory state caused by exposure to air pollution has been shown to alter microglial function and neuronal development, as well as axonal myelination, thus affecting several processes of neurodevelopment that have been linked to SCZ pathogenesis.

## The Gut-Brain Axis in SCZ

The CNS communicates bi-directionally with the gastrointestinal (GI) system to maintain homeostasis, for instance by regulating hunger and digestion processes at steady state (Konturek et al., 2004). There has been extensive study of the reciprocal gut and CNS interactions, which communicate through the enteric nervous system and vagus nerve, and via alternative pathways involving the immune and neuroendocrine systems (Sudo et al., 2004; Sampson et al., 2016; Singh et al., 2016) or through direct secretion by gut microbes of neurotransmitters (Yano et al., 2015) and metabolites (De Vadder et al., 2014; Sherwin et al., 2019). However, the importance of the gut in mediating brain function and behavior was ignited by the discovery that germ-free mice, which are devoid of microorganisms, have heightened stress responses (Sudo et al., 2004). In more recent work, the microbiota has been shown to influence complex behaviors such as social behavior, depression, and anxiety which are directly relevant to SCZ and other neuropsychiatric disorders (Desbonnet et al., 2014; Sherwin et al., 2019). Additionally, 19% of people with SCZ are comorbid for irritable bowel syndrome, which has a known inflammatory etiology (Gupta et al., 1997) compared to an occurrence rate of only 2.5% in the general population. Studying the role of the microbiota in disease states is challenging since it is highly sensitive to environmental changes. Therefore, most of the environmental risk factors for SCZ also impact the microbiota (Franklin and Ericsson, 2017), making it difficult to determine causation. However, recent work suggests a causative role for the microbiota in neuropsychiatric disorders and highlights the role of the immune system in linking the brain and gut in pathological conditions (Castro-Nallar et al., 2015; Yolken et al., 2015; Schwarz et al., 2018; Zheng et al., 2019; Zhu et al., 2019).

The microbiota not only plays a key role in regulating host metabolism but also modulates inflammatory responses and neural function. Germ-free mice have multiple deficits in nervous system function including heightened hypothalamic-pituitary-adrenal (HPA) axis responses (Sudo et al., 2004), altered anxiety-like behaviors (Neufeld et al., 2011), increased motor activity (Diaz Heijtz et al., 2011), and impaired memory (Gareau et al., 2011), and social behaviors (Desbonnet et al., 2014). In healthy individuals, increased HPA axis function is generally associated with a suppression of subclinical inflammation due to the anti-inflammatory properties of glucocorticoids (Barnes, 1998). However, the ability of cortisol to suppress inflammation might be altered in SCZ, instead correlating with increased inflammation evident by an increase in IL-6 (Chiappelli et al., 2016).

The microbiota of people with SCZ has been found to contain more of the bacterial species *Lactobacillus* compared to healthy controls, and levels of this bacterium correlate with psychosis severity (Castro-Nallar et al., 2015; Yolken et al., 2015; Schwarz et al., 2018). In a recent study, gut microbiota from SCZ patients was transferred into germ-free mice to test whether SCZ-relevant behavioral phenotypes were transmissible via their gut microbiome. Germ-free mice receiving fecal transplants from these patients had lower levels of glutamate and higher levels of glutamine and GABA in the hippocampus, and these mice exhibited locomotor hyperactivity and decreased anxiety-like and depressive-like behaviors, as well as increased startle responses relative to control mice that received fecal transplants from healthy subjects (Zheng et al., 2019). However, SCZ patients in this study were receiving antipsychotic treatment, which has been shown to alter the gut-microbiome (Bretler et al., 2019) so this could be a confounding effect. Transplantation of the gut microbiome from drug-free individuals with SCZ into antibiotic-treated mice caused SCZ-related phenotypes such as impaired learning and memory as well as increased psychomotor behaviors, while also leading to increased PFC dopamine and hippocampal serotonin levels compared to mice receiving microbiota transplants from healthy controls (Zhu et al., 2019), suggesting drug-independent effects of the gut-microbiome in SCZ.

Microbes are able to produce or aid in the production of multiple neurotransmitters, including serotonin, dopamine and GABA, but it is still unclear how the gut production of these neurotransmitters affects CNS function (Yano et al., 2015; Strandwitz, 2018). Additionally, gut microbiome transplantation or treatment with probiotics has been shown to, at least partially, reverse MIA-associated phenotypes in rodents, including deficits in anxiety-like, stereotypic and sensorimotor behaviors (Hsiao et al., 2013). The reversal of these phenotypes seems to be mediated through the normalization of gut permeability and microbe dysbiosis (Hsiao et al., 2013), suggesting that the gut microbiota can directly modulate immune responses even between a dam and its embryo. This is not surprising given that the microbiota has a well-studied role in inducing and maintaining the function of the host immune system.

The gut microbiome can affect the integrity of the blood-brain barrier (BBB), which facilitates increased neuroinflammation. The presence of gut microbes is necessary for the proper formation of the BBB during early development. Mice from germ-free dams have disrupted BBB maturation, which is evident by decreased tight junction expression both prenatally and postnatally. The hyperpermeability of the BBB in germ-free mice persists into adulthood, but can be rescued by microbiota transplantation from controls or through the administration of bacteria that produce short chain fatty acids (SCFAs) (Braniste et al., 2014), which are known to have anti-inflammatory effects and promote BBB integrity (Hoyles et al., 2018). As mentioned previously, the gut plays an important role in the differentiation of Th17 cells. Interestingly, the gut also promotes the infiltration of Th17 cells into the brain through the meninges where these cells secrete IL-17, which further promotes immune cell infiltration [reviewed in Cipollini et al. (2019)]. BBB endothelial cells express TLRs and therefore are able to respond to gut microbe components such as LPS, which can alter tight junction expression and promote immune cell infiltration into the CNS (Tang et al., 2017). The BBB and microbiome are both disrupted in SCZ; this works thus highlights the potential for crosstalk between these systems that might act synergistically to further contribute to neuroinflammation in SCZ.

Gut microbes produce metabolites that can cross the BBB and inhibit the function of mitochondria in the CNS (Hulme et al., 2020). A decrease in mitochondria density and altered structure has been observed in post-mortem SCZ brain tissue across multiple regions including the anterior cingulate cortex (Flippo and Strack, 2017; Roberts, 2017). This finding raises the intriguing possibility that gut microbe metabolites can contribute to SCZ pathology. While the identity of the CNS cell(s) affected by gut metabolites remains unclear, the dysfunction of mitochondria in microglia has been shown to alter cytokine production and inflammatory responses in the brain [reviewed in Culmsee et al. (2018)]. MIA, which increases the risk for SCZ, has been shown in mice to alter the structure of mitochondria in a disease-associated microglial subtype known as dark microglia, among the hippocampus (Hui et al., 2018). Taken together, these studies suggest that there is extensive interplay between risk factors for SCZ, such that signaling from the gut-brain axis and exposure to an early immune insult can alter the function of microglia and CNS mitochondria. Future studies could aim to target the gut microbiome to dually control BBB integrity and reduce neuroinflammation.

Gut microbiota dysbiosis can alter the maturation and function of microglia in the CNS, thus contributing to neuroinflammation (Erny et al., 2015; Thion et al., 2018). Germ-free mice have microglia with an immature morphology and gene expression profile in adulthood (Erny et al., 2015), suggesting that the microbiota impacts the maturation of microglia. The absence of microbes was found to not only affect microglial function but also impair innate immune responses, which were partially recovered by colonization with a more complex microbiome or by supplementation with SCFAs, which are a by-product of certain gut microbes (Erny et al., 2015). SCFAs might affect CNS function through their interactions with BBB endothelial cells (Braniste et al., 2014) or directly with the CNS considering that they do not require receptors to bypass the BBB (Frost et al., 2014). The lack of SCFAs could additionally lead to increased peripheral and central inflammation considering their well-known anti-inflammatory functions (Vinolo et al., 2011; Li M. et al., 2018).

Microglia also show sex-dependent differences in response to gut microbe sterility. Microglia from germ-free male mice displayed altered expression of immune genes and a more immature phenotype at juvenile stages whereas microglia from female mice were more affected in adulthood (Thion et al., 2018). These findings suggest that the maternal microbiome can regulate microglial function in the offspring brain (Thion et al., 2018), notably in the context of MIA exposure (Kim et al., 2017; Shin Yim et al., 2017), in a sexually dimorphic manner. Sex differences in microglial response to microbiome challenges are intriguing as they could partially explain the earlier onset of SCZ in males compared to females (Ochoa et al., 2012). MIA models also display sexual dimorphism in microglial properties and behavioral outcomes (Hui et al., 2018). However, much work is needed to understand whether microglia-induced sex differences are present in SCZ.

Without a doubt, the gut microbiome influences the development and maintenance of the immune and nervous systems, with significant crosstalk. In the context of SCZ, the metabolites and diversity of gut microbes may impact multiple disease symptoms. The microbiome links multiple risk factors for SCZ, including stress responses, by promoting immune activation and BBB disruption. Innate immunity of the brain, including microglial function, is sensitive to gut dysbiosis, making the gut microbiota an interesting target in SCZ. Probiotics and microbiome transplants should be further explored to improve symptom severity in people with SCZ. Additionally, precautionary steps could be taken in pregnant mothers to improve diversity of gut microflora, considering its profound impact on brain development. Future work should further explore the role of SCFA-producing microbes, considering that they exert anti-inflammatory effects and improve brain function and behavior. Taken together, gut microbes are positioned to alter immune responses to environmental challenges by regulating neuronal function, behavior, and microglial responses, all of which are altered in SCZ.

## MIA Enhances Risk for SCZ by Altering Microglial Function

It has become increasingly clear that immune challenges occurring during pregnancy increases offspring risk for varied neurodevelopmental and neuropsychiatric disorders, including SCZ. Specifically, maternal exposure during pregnancy to bacterial (Sørensen et al., 2009) or viral infections such as influenza, rubella or herpes (Pearce, 2001; Brown and Derkits, 2010) leads to lasting changes in offspring brain function and behavior (Estes and McAllister, 2016). Maternal infection has been extensively studied using animal models of MIA, which have provided a substantial amount of causative evidence for how early immune insults disrupt brain development and function (Knuesel et al., 2014; Estes and McAllister, 2016). MIA can be induced by exposing pregnant dams to immunogens that mimic an infection. The most common immunogens used to model MIA include polyinosinic:polycytidylic acid [poly(I:C)] and LPS which mimic viral or bacterial infection, respectively. These agents elicit immune responses that enable cytokines to pass through the placental barrier, activating placental and embryo macrophages, and leading to increased inflammation in the developing offspring (Wu et al., 2017). Although work is needed to normalize MIA protocols, particularly on the temporal level, and to understand the variability in reported results (Kentner et al., 2019), this animal model has provided insight into how maternal infection enhances the risk for various disorders. Here, we focus on progress that has been made in understanding prenatal immune challenges in mice and humans.

MIA impacts brain function in a circuit-specific manner and interacts with other risk factors for SCZ. These early immune insults can elicit a vast array of phenotypes in mice that are relevant to SCZ and ASD, including abnormalities in ultrasonic vocalization and sociability, increased repetitive behaviors, motor dysfunction, and deficits in sensorimotor gating and cognitive abilities such as working memory (Knuesel et al., 2014; Fernández de Cossío et al., 2017; Pendyala et al., 2017; Shin Yim et al., 2017). Some of these behavioral effects are sex-dependent (Haida et al., 2019). MIA-induced behaviors were accompanied by changes in specific brain areas such as altered hippocampal volume and cortical thickness, and changes in synaptic density and proteins (Estes and McAllister, 2016; Fernández de Cossío et al., 2017), which are also observed in SCZ (Glantz and Lewis, 2000; Hui et al., 2018; Onwordi et al., 2020). Alterations in amygdala-cortical circuitry have been implicated in SCZ (Benes, 2010) and a recent study showed that MIA enhances glutamatergic neurotransmission between these circuits by increasing synaptic strength in the exposed offspring (Li Y. et al., 2018). An exciting development in this field showed that MIA-induced deficits in neurodevelopment depend on inflammatory signaling through the maternal microbiome (Kim et al., 2017). MIA via LPS also disrupts BBB function by increasing its permeability, thus promoting neuroinflammation (Estes and McAllister, 2014; Simões et al., 2018). However, there is also evidence for no change in BBB permeability after MIA induced via poly(I:C) in mice (Garay et al., 2013), suggesting immunogen-dependent effects. These differences also emphasize the variability of MIA animal models and the need for experimental standardization.

Given that microglia are the primary innate immune cells of the brain, they provide rapid responses to immune insults and are greatly affected by systemic inflammation. MIA exerts its effects on neurodevelopment largely by disrupting microglial function and by priming them for altered responses later in life. Changes in the density of microglia are found in early postnatal MIA offspring in multiple cortical and subcortical regions including the anterior cingulate cortex, striatum and hippocampus (Zhang et al., 2018). Microglial involvement in MIA effects is evident through an increase in cytokine and chemokine signaling, in mouse hippocampus and basal forebrain, during late fetal development in response to either LPS (Schaafsma et al., 2017) or poly(I:C) (Pratt et al., 2013). A recent study showed that an MIA mouse model induced at embryonic day 9.5 with poly(I:C) led to an increased density of a pathological microglial subtype, called dark microglia, in the hippocampus of male versus female offspring (Hui et al., 2018). Dark microglia are almost exclusively observed in disease states or in aged animals, and exhibit greater levels of oxidative stress and hyper-ramified processes in closer proximity to synapses than typical microglia (Bisht et al., 2016). These studies highlight the ability of MIA to alter microglial state and function.

**FIGURE 2:**
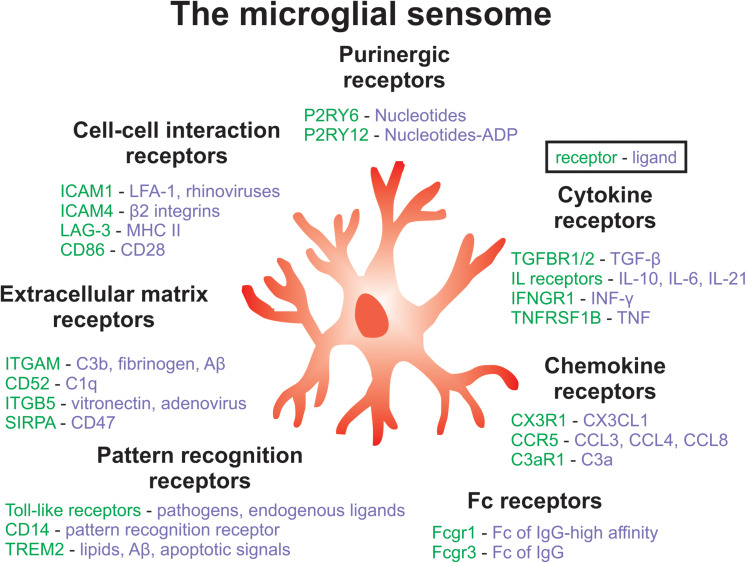
Key components of the microglial sensome associated with SCZ. The microglial sensome is a group of receptors and proteins that allow microglia to sense and respond to their changing environment, facilitating the diverse roles of microglial as well as their complex interactions with multiple cell-types in the brain. Components of the microglia sensome can be categorized to include purinergic, cytokine, Fc, pattern recognition, extracellular matrix, and cell-cell interaction receptors and ligands, among others not listed here. Future work could aim at targeting the microglial sensome to normalize microglial function in SCZ.

Moreover, MIA in mice alters the transcriptome and phagocytic activity of microglia in offspring (Mattei et al., 2017). Specifically, hippocampal microglia from male poly(I:C) mice displayed a downregulation of genes that encode cell surface receptors associated with phagocytosis (*P2ry6, Sirpa, Siglece, Cx3cr1, Fcgr1, Itgav)* (Mattei et al., 2017). These receptors are important components of the microglial ‘sensome’, which contribute to the regulation of microglia-neuron interactions and are important for the engulfment of neuronal material (Mattei et al., 2017; Hickman and El Khoury, 2019; Figure 2). Inflammatory abnormalities, such as increased levels of SERPINA3, TNFα, IL-1β, IL-6, and IL-6ST, have been observed in the ventral midbrain in post-mortem SCZ tissue, and these results were also replicated in an MIA mouse model (Purves-Tyson et al., 2019). Importantly, these differences in immune markers from SCZ tissue could be accounted for by a subset of cases, including about 45% of high inflammatory cases. The ventral midbrain houses the majority of dopamine-releasing neurons in the brain, therefore MIA might contribute to SCZ pathology by disrupting immune-mediated wiring of dopaminergic circuits (Purves-Tyson et al., 2019). These findings are important because they link SCZ-associated neuroinflammation to dopaminergic abnormalities, which are a hallmark of this disorder.

Multiple studies targeting microglial signaling pathways were able to reverse MIA-associated neuropathology, suggesting that microglia are the main culprit in inducing neurological dysfunction in response to immune challenges. For example, a study that targeted colony stimulating factor 1 receptor (CSF-1R), which plays a role in microglial proliferation, was successful in reversing some MIA-induced phenotypes (Ikezu et al., 2020). Depleting and repopulating microglia by inhibiting CSF-1R was protective in mice exposed to poly(I:C) prenatally (Ikezu et al., 2020). Specifically, once the microglial population was renewed, not only were the deficits in repetitive and social behaviors reversed, but normal neuronal connectivity and microglia-neuron interactions were also restored (Ikezu et al., 2020). Another successful approach to restore typical microglial function targeted the peroxisome proliferator-activated receptor gamma (PPARγ) signaling pathway. PPARγ signaling is activated by fatty acids and reduces myeloid cell-induced inflammation via suppressing their production and/or secretion of inflammatory molecules (Bernardo and Minghetti, 2006). Agonists of PPARγ have been found to be protective in the context of MIA by inhibiting microglial expression of pro-inflammatory cytokines and surface antigens (Bernardo and Minghetti, 2006), suggesting that targeting microglial PPARγ signaling could be beneficial in offspring exposed to MIA (Zhao et al., 2019). In support of this, a recent study showed lower serum levels of PPARγ in patients with SCZ while levels of this biomarker decreased further with disease progression (Yüksel et al., 2019). Treatment with minocycline, a broad-spectrum anti-inflammatory and antibiotic drug that generally restores microglial functions, also reversed changes in microglial transcriptome and phagocytic activity in mouse offspring exposed to MIA (Mattei et al., 2017). Lastly, there is evidence that deep brain stimulation in rats can prevent some of the behavioral deficits associated with MIA specifically by reducing microglial pro-inflammatory responses (Hadar et al., 2017). Taken together, these data suggest that microglia play a critical role in MIA-induced brain dysfunction and that targeting microglia is a potential therapeutic approach to reverse MIA-induced phenotypes.

MIA is a risk factor for SCZ that depends on maternal immune signaling relayed to the fetal brain through the placenta. Maternal gut microorganisms have been found to play an important role in MIA-mediated deficits. A ground-breaking study showed that MIA phenotypes in exposed offspring are dependent on the presence of segmented filamentous bacteria in the maternal gut which promote Th17 cell differentiation, leading to increased IL-17a production (Kim et al., 2017). MIA phenotypes, including deficits in cortical development and behavioral abnormalities, were dependent on gut microbiome-mediated increases in IL-17a (Kim et al., 2017; Shin Yim et al., 2017). These data show that maternal microbe-induced immune signaling impacts fetal brain development with long-term consequences and that prenatal inflammatory insults can prime the gut-immune-brain axis, thus leading to altered CNS responses to immune challenges later in life.

MIA is an important model that has increased our understanding of how immune insults occurring during embryonic development can alter brain development. Although there is variability in data obtained using mouse models of MIA, notably due to differences in immunogen manufacture (molecular weight, endotoxin contamination, etc.), timing of immunogen administration, dosage, route of administration, housing conditions, timing of cage cages and mouse strain used (Careaga et al., 2018; Kentner et al., 2019; Kowash et al., 2019), understanding what causes these differences could aid in understanding the mechanisms underlying vulnerability versus resiliency to MIA (Meyer, 2019). In humans, only a subset of pregnant mothers who are exposed to a viral or bacterial infection have offspring who later develop SCZ (Estes et al., 2019). This is to be expected since immune activation is only one of the many risk factors for SCZ. Therefore, the variability in mouse models of MIA might be exploited to elucidate why certain sub-populations of individuals are at greater risk for SCZ. Since some mouse strains are resilient to MIA, the genetic differences between mouse strains could be used to identify protective versus susceptibility genes (Schwartzer et al., 2013). Overall, future work aimed at understanding such variability will likely be valuable in discovering only a subset of subjects are vulnerable to MIA.

It is interesting that MIA is a risk factor for both SCZ and ASD, since some of the neurological deficits observed in these disorders appear to be opposing. For example, SCZ is characterized by a significant loss of gray matter resulting in hypoconnectivity between the anterior hippocampus and PFC (Vita et al., 2012; Blessing et al., 2020), on which the neonatal ventral hippocampal lesion rodent model of SCZ is based (Joseph et al., 2018), whereas ASD is associated with hyperconnectivity (Supekar et al., 2013). How could the same risk factor play a role in such opposing phenotypes? We propose that the underlying genetic background and the time of exposure are important factors that determine the effects that MIA exerts on brain development. For example, SCZ is associated with genetic variation in the *C4* gene that led to enhanced *C4* expression (Sekar et al., 2016) whereas *C4*, *C3*, and *C1q* were found to be downregulated in ASD (Fagan et al., 2017). Differences in certain genes, such as complement genes, which have an established role in synaptic pruning (Stevens et al., 2007; Schafer et al., 2012; Sekar et al., 2016; Comer et al., 2020), could explain how MIA differentially contribute to disease phenotypes. Alternatively, the expression of *TLR3* and *TLR4*, which directly respond to poly(I:C) and LPS (Lu et al., 2008; Zhou et al., 2013), respectively, could differ between mouse strains with varying susceptibility to MIA and in humans predisposed to different NDDs. Lastly, it is not clear how recently emerging viruses, such as SARS and MERS coronaviruses, might contribute to NDDs (Gretebeck and Subbarao, 2015; Fauci et al., 2020). It is also unknown whether the severe acute respiratory syndrome coronavirus 2, which caused the COVID-19 pandemic, leads to lasting consequences on brain development and behavior while preliminary data suggest that passive transfer of antibodies from mother to embryo is possible (Zeng et al., 2020).

## Stress-Induced Inflammation and Microglial Dysfunction

**FIGURE 3:**
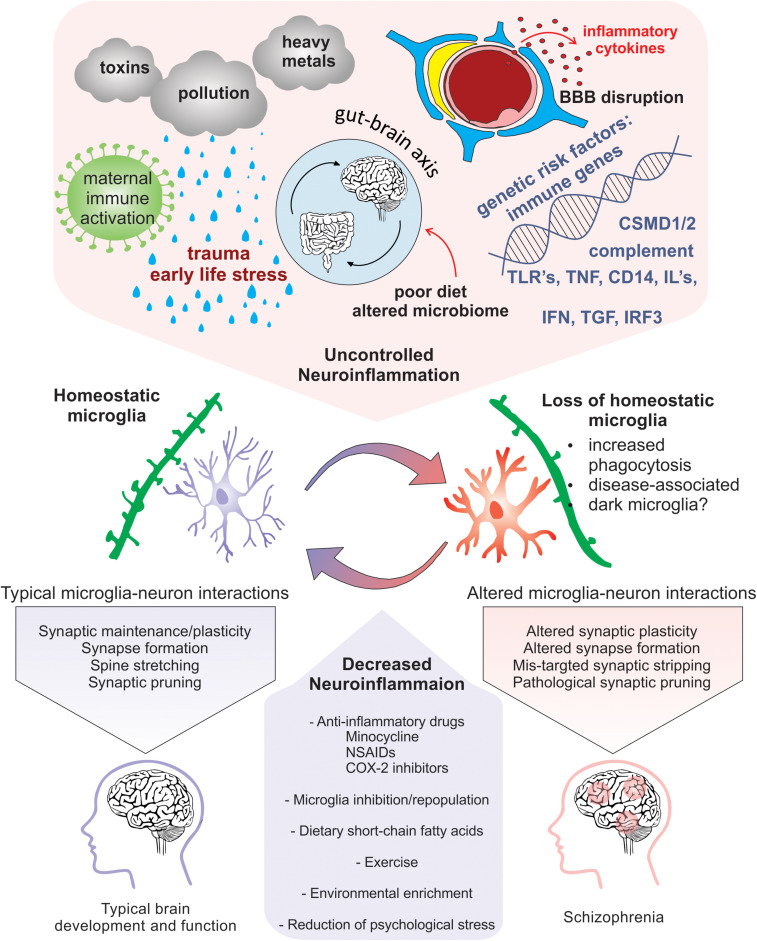
Neuroinflammation-induced changes in microglia that are implicated in SCZ pathogenicity. Risk factors for SCZ that alter microglial function and enhance neuroinflammation include pollution, stress, nutrition induced gut-brain axis dysbiosis, viral infection, maternal immune activation, genetic predisposition, and cytokine secretion. Homeostatic microglia perform their immune sentinel role by interacting with neurons to guide circuit wiring during development. In an increased inflammatory milieu, loss of microglial homeostasis perturbs microglia-neuron interactions that may cause altered plasticity due to pathogenic synaptic formation, synaptic stripping, and pruning. Therapeutic approaches that promote homeostatic microglia through the reduction of neuroinflammation via anti-inflammatory drugs, microglial inhibition and repopulation, improved nutrition, environmental enrichment, and prevention of psychological stress could be potentially exploited to limit exacerbation of SCZ.

Exposure to psychological stress or traumatic life events prenatally and during childhood or adolescence results in an increased risk for SCZ (Weinstock, 2008; Read et al., 2009; Kessler et al., 2010; Holtzman et al., 2013). Specifically, during critical periods of development, certain stressors, such as physical or mental abuse, socioeconomic disadvantage, living in an urban environment and neglect, all confer greater risk for SCZ (McGrath et al., 2004; Quidé et al., 2017; Popovic et al., 2019). Additionally, people with SCZ have altered physiological responses and increased vulnerability to stressful stimuli (Schifani et al., 2018). Thus, increased exposure and vulnerability to psychosocial stress, especially during critical periods of brain development, represents a significant challenge. However, cellular and molecular mechanisms that link early life stress (ELS) with increased risk for SCZ are still unclear. Nevertheless, evidence suggest that psychosocial stressors contribute to SCZ pathology by in part increasing neuroinflammation (Figure 3). A unified review was recently published focusing on the relationship between childhood trauma and psychosis, integrating results of epidemiological, clinical, neuropsychological and biological studies (Misiak et al., 2017).

Individuals with SCZ have altered physiological stress responses (van Venrooij et al., 2012; Schifani et al., 2018; van Leeuwen et al., 2018). Exposure to stress stimulates the sympathetic nervous system causing the secretion of epinephrine and norepinephrine, resulting in increased HPA axis function which leads to the release of stress hormones, such as cortisol, into the blood [reviewed by Chrousos (2009)]. These stress hormones alter an organism’s physiology to promote activities that combat the stressor, such as increased cardiac function and glucose availability, while decreasing less urgent processes including digestion, reproduction, and immune function (Chrousos, 2009). In healthy individuals, cortisol led to the suppression of adaptive immunity and an increase in innate immunity due to the effects of glucocorticoids on inflammation (Barnes, 1998). Although cortisol has some anti-inflammatory effects, its ability to regulate inflammatory responses is altered in SCZ. In healthy individuals, an acute stressor led to increased salivary levels of cortisol and a decrease in IL-6; however, in individuals with SCZ, an increase in cortisol was shown to be accompanied by an increase in IL-6 (Chiappelli et al., 2016). Additionally, chronic and ELS, which are risk factors for SCZ, are linked to increased immune activation (Chiappelli et al., 2016), as well as abnormal sensitivity and levels of glucocorticoids and their receptors (Webster et al., 2002; Sinclair et al., 2012; do Prado et al., 2017), disrupting the ability of cortisol to regulate inflammation (Miller and Chen, 2010). In this sense, stress-induced release of cortisol might increase inflammatory responses in people with SCZ instead of having anti-inflammatory effects such as seen in healthy individuals.

Although multiple studies have found an increase in HPA axis function in people with SCZ (Walder et al., 2000; Mondelli et al., 2010a; Chiappelli et al., 2016), others have reported a decrease in cortisol levels compared to controls in response to a stressor (Ciufolini et al., 2014; Lange et al., 2017; Glassman et al., 2018). The inconsistencies between these findings could be due variation including differences in stressor intensity, duration, time point of exposure (Lange et al., 2017), or administration of antipsychotics, which have been shown to alter cortisol stress responses (Houtepen et al., 2015). Despite these discrepancies, HPA axis dysfunction has been observed in first-episode psychosis prior to antipsychotic treatment (Ryan et al., 2004; Mondelli et al., 2010b; Mondelli et al., 2010a). Additionally, recent work has shown that regardless of differences in cortisol responses to acute stressors among people with SCZ, those with decreased cortisol responses to social stress had lower measures of social functioning (Tas et al., 2018). Therefore, understanding differences in cortisol responses and its relationship to immune function in SCZ could provide insight into the role of psychosocial stress on disease progression.

Prenatal psychological stress is associated with an increased risk of SCZ (Kofman, 2002; Weinstock, 2008; Pugliese et al., 2019). In mice, prenatal stress increased placental expression of several pro-inflammatory genes including *IL-6*, *IL-1B*, and *TNF*α specifically in males, and these changes were partially rescued by maternal administration of a non-steroidal anti-inflammatory drug (Bronson and Bale, 2014). Additionally, studies in mice have shown that male offspring exposed to prenatal stress displayed behavioral deficits including anhedonia and changes in stress responses that coincided with altered placental gene expression in males but not females, affecting *PPAR*α, the growth factor *IGFBP-1*, hypoxia-inducible factor 3a (*HIF3*α), and glucose transporter *GLUT4*, all of which have been implicated in immune system function (Mueller and Bale, 2008). Importantly, the placenta is a regulator of maternal-fetal immune initiation in offspring [reviewed in Hsiao and Patterson (2012)] and this interaction appears to be crucial given that prenatal dysregulation of the immune system can lead to altered immune responses postnatally (Bilbo and Schwarz, 2009; Pedersen et al., 2018). Maternal restraint stress resulted in offspring with altered microglial morphology and density in the cortical plate at embryonic stages and in neocortex at adulthood, and these prenatal-induced changes were reversed by blocking IL-6 (Gumusoglu et al., 2017), confirming that increased maternal expression of IL-6 can cause neuroinflammation in embryos by crossing the placenta (Dahlgren et al., 2006). Nevertheless, the role of maternal stress-induced inflammation and the specific involvement of the placenta in mediating its consequences are not fully understood.

ELS, such as childhood abuse or neglect, is a major risk factor for SCZ, however the mechanisms by which ELS induces changes in neuronal circuitry is not clear. Mounting evidence suggests that dysfunction of the immune system and microglia, especially, can contribute to brain miswiring and behavioral deficits after ELS (Na et al., 2014; Johnson and Kaffman, 2018). In humans and mice, ELS increases multiple blood pro-inflammatory markers including CRP, IL-1β, IL-6, IL-8, TNF-α (Hepgul et al., 2012; Marsland et al., 2017; Réus et al., 2017) while suppressing the anti-inflammatory cytokine IL-10, leading to depressive-like behaviors in mice (Réus et al., 2017). In line with these findings, ELS resulted in altered microglial gene expression, density, morphology and phagocytic activity during maturation in particular brain regions including the mPFC, striatum, anterior cingulate cortex and hippocampus (Cohen et al., 2016; Delpech et al., 2016; Bollinger et al., 2017; Wang et al., 2017; Banqueri et al., 2019; Réus et al., 2019). Chronic stress also altered microglial function by activating the P2X7 receptor, which induced the NLRP3 inflammasome thus increasing levels of mature IL-1β within the brain (Pan et al., 2014; Yue et al., 2017).

Since microglia play vital roles in brain development and homeostasis including neurogenesis, synaptic formation and elimination (Salter and Beggs, 2014; Hong et al., 2016; Tay et al., 2017b), their dysfunction could explain some of the neurological deficits observed after exposure to stress. Studies using RT-PCR from isolated microglia show that steroid hormone receptors, such as the glucocorticoid receptor, are abundant in microglia (Sierra et al., 2008), suggesting the possibility that stress could directly impact microglial function through glucocorticoid signaling. Indeed, a line of evidence suggests that stress can impact microglial proliferation, while blocking corticosterone synthesis or glucocorticoid receptor activity restored normal microglia density in mice (Nair and Bonneau, 2006; Duque and Munhoz, 2016). There is evidence that stress later in life can also induce changes in microglia, especially when these cells are primed by an environmental insult either prenatally or during early postnatal development (Catale et al., 2020). For instance, mice that were susceptible to repeated social defeat had microglial transcriptomes that were enriched for markers of phagocytosis, pro-inflammatory responses and reactive oxygen species compared to mice that were either resistant or not exposed to stress (Lehmann et al., 2018). Additionally, mice that were sensitive to repeated social defeat showed an increase in markers for extracellular matrix remodeling and BBB leakage, which coincided with an enhanced permeability of the BBB to a fluorescent tracer, and correlated with increased microglial phagocytosis of neuronal material (Stankiewicz et al., 2015; Lehmann et al., 2018). Additionally, microglial depletion by the CSF1R antagonist PLX5622 in a repeated social defeat mouse model protected against the behavioral abnormalities and prevented an increase in reactive oxygen species in the mPFC, nucleus accumbens and paraventricular nucleus (Lehmann et al., 2019). Together, these data support that microglia play a vital role in stress-induced neuropathology by becoming more phagocytic, inducing the inflammasome and engulfing neuronal material.

Psychosocial stress might be more preventable than the other risk factors for SCZ. Reducing psychosocial stress in expecting mothers and young children or combating stress with exercise, nature exposure, yoga, or therapy could be used in individuals at risk for or diagnosed with SCZ (Entringer et al., 2009; Vancampfort et al., 2011; Brannigan et al., 2019). Some lines of evidence show that environmental enrichment can protect against or reverse many effects of stress, including ELS, by rescuing behavioral phenotypes, inflammatory responses, microglial function, and oxidative stress, notably in the mPFC (do Prado et al., 2016; McCreary and Metz, 2016; Dandi et al., 2018; González-Pardo et al., 2019), a region implicated in SCZ (Glantz and Lewis, 2000; Barch et al., 2001). However there is conflicting evidence concerning the ability of environmental enrichment to rescue these phenotypes in severe cases of ELS (Mackes et al., 2020). Alternatively, future studies could determine if treatment with anti-inflammatory medications can protect against stress-induced neuroinflammation since microglial depletion has been shown to be protective (Lehmann et al., 2019).

## How the Peripheral Immune System Gains Access to the CNS in SCZ

The link between BBB dysfunction and SCZ was first established when epidemiological studies revealed that about two-thirds of SCZ cases are diagnosed with comorbid conditions associated with deficits in endothelial cell function, such as metabolic syndrome and cardiovascular disease (Israel et al., 2011; Burghardt et al., 2014). Capillary wall endothelial cells form tight junctions with one another and are an integral component of the BBB along with pericytes, astrocytic endfeet, microglia, and the extracellular matrix that forms the basement membrane (Lassmann et al., 1991; Abbott et al., 2010; Bisht et al., 2016; Joost et al., 2019). The BBB restricts the passage of molecules between the blood and the brain to protect sensitive neural tissue from pathogens and immune molecules while allowing the passage of vital molecules such as glucose (Abbott et al., 2010). This allows the BBB to isolate the brain from peripheral immune responses; however, it has become increasingly clear that in pathological states the ability of the BBB to isolate the CNS from harmful immunological responses is disrupted (Bechter et al., 2010; Najjar et al., 2017).

Claudin-5, expressed in brain endothelial cells, forms a major component of the BBB barrier-forming tight junctions (Morita et al., 1999; Greene et al., 2019). Claudin-5 maps to a region on chromosome 22 where small deletions cause the 22q11 deletion syndrome, which is found in 30% of SCZ cases (Murphy, 2002; Motahari et al., 2019). People with this syndrome are haploinsufficient for claudin-5 and have increased odds of developing SCZ (Fiksinski et al., 2018; Greene et al., 2018). A recent study showed that during acute versus chronic inflammation, levels of claudin-5 are differentially expressed (Haruwaka et al., 2019). It is still unknown if microglial phagocytosis of tight junctions is also involved in SCZ, although this finding suggests that BBB dysfunction could be mediated through a decrease of molecules involved in tight junctions or BBB permeability.

Indeed, post-mortem mPFC tissue from SCZ individuals show changes in the endothelial cell gene expression of molecules involved in tight junctions and BBB permeability. People with SCZ can be divided into subgroups based on their extent of brain and serum inflammatory markers (Fillman et al., 2016). Cases of SCZ that have higher serum pro-inflammatory markers, which include about 40% of affected people (Fillman et al., 2016), also have greater gray matter loss in the mPFC, which is thought to underlie multiple symptoms of SCZ (Zhang et al., 2016). Compared to healthy controls, SCZ cases, especially high-inflammatory cases, have increased expression of the intercellular adhesion molecules ICAM-1 and VCAM-1 in endothelial cells from the PFC (Kavzoglu and Hariri, 2013; Cai et al., 2018; Nguyen et al., 2018). ICAM-1 and VCAM-1 interact with receptors on leucocytes to allow monocyte infiltration into the brain (Hermand et al., 2000). In endothelial cell cultures, ICAM-1 expression can be induced in a dose-dependent manner by the pro-inflammatory cytokine IL-1β (Cai et al., 2018). ICAM-1 expression has also been found to correlate with the expression of the macrophage marker CD163, and CD163-positive macrophages were found in close association with neurons in the frontal cortex of high-inflammatory SCZ cases (Cai et al., 2018). In this study, proteins that form endothelial cell tight junctions, including cadherin-5 (CDH5) and occluding (OCLN), were also upregulated in the frontal cortex (Cai et al., 2018), which highlights a compensatory mechanism to regain BBB integrity. Conversely, multiple studies have a found a decreased expression of CDH5 in the PFC of SCZ individuals, while genetic knockdown of CDH5 in mouse PFC led to BBB disruption and changes in behavior including deficits in learning, memory, sensorimotor gating, and anxiety-like behavior (Nishiura et al., 2017; Greene et al., 2018). The expression of tight junction genes could differ depending on the time point during SCZ progression, such that compensatory mechanisms could be elicited in later disease stages. In addition, the conflicting evidence for a leaky BBB in SCZ suggest that the BBB is compromised in only a subset of SCZ cases. The finding of subgroups of people with SCZ showing variable levels of systemic inflammation support this hypothesis. Together, these findings reveal the importance of studying subgroups of SCZ patients, based on systemic inflammation, to gain a more comprehensive understanding of the disease pathogenesis.

In addition to endothelial cells, pericytes and astrocytes have also been implicated in BBB dysfunction during systemic inflammation (Fabry et al., 1993; Nishioku et al., 2009; Chen et al., 2017; Banks et al., 2018). There is some evidence that pericytes can exit the perivascular space in response to LPS-induced inflammation in mice, while the extent of pericyte detachment correlated with microglial reactivity (Nishioku et al., 2009). Pericytes secrete cytokines, including IL-1 and IL-6, which are capable of disrupting endothelial cell tight junctions (Fabry et al., 1993). Disruption of the BBB in several mouse models of neuropsychiatric or inflammatory diseases has been shown to affect microglial function, while dynamic neuroimmune interactions were described at the BBB in both health and diseased sates (Merlini et al., 2012; Borjini et al., 2019; Haruwaka et al., 2019). Although causal evidence is needed, multiple studies have found that microglial reactivity worsens BBB integrity in pathological states and that administration of the anti-inflammatory drug minocycline can improve BBB function (Yenari et al., 2006; da Fonseca et al., 2014; Shigemoto-Mogami et al., 2018). More work is still needed to understand whether or how the interplay between BBB dysfunction and microglia abnormalities contribute to the pathogenesis of SCZ. Complex cytokine signaling between the pericytes, endothelial cells, astrocytes and microglia is crucial for the development and maintenance of BBB integrity (Chen et al., 2017; Banks et al., 2018). Lastly, it was suggested that PFC hypoconnectivity in SCZ might result from altered blood flow regulated by pericytes, together with abnormalities in the structures of capillaries and astrocytic end feet (Uranova et al., 2010). As such, understanding the complex interactions between cell-types of the neurovascular unit and how they might be altered in response to inflammation in SCZ will likely be important.

Abnormal activity in multiple brain networks and regions are observedin SCZ (Uhlhaas, 2013). There is clear evidence that excitatorycircuits are altered in SCZ (Glantz and Lewis, 1997, 2000; Uhlhaas, 2013). Blockade of *N*-methyl-D-aspartate receptors (NMDARs) in healthy subjects leads to psychotic symptoms and cognitive deficits that resemble those observed in SCZ (Balu, 2016). Additionally, both mRNA and protein levels of the NMDA subunits NR1 and NR2C are decreased in post-mortem SCZ brain tissue (Weickert et al., 2013). Recent evidence suggests that NMDAR function might be inhibited in SCZ by autoantibodies, which are produced against an organism’s own tissue and are implicated in autoimmune disorders such as lupus (Becker et al., 2019). Circulating autoantibodies against glutamate and NMDARs were found to be present in approximately 20% of psychotic SCZ patients (Jézéquel et al., 2017). An increased BBB permeability might alter neuronal function by allowing the entry of autoantibodies against NMDARs into the brain, which have been shown in mouse models and neuronal culture experiments to suppress glutamatergic activity by altering the organization of NMDARs and their anchoring molecule ephrin-B2 (Kayser and Dalmau, 2016; Jézéquel et al., 2017; Kannan et al., 2017). Studies that interrogate specific cell-type and neural circuit responses will allow greater understanding of the impact of BBB permeability on brain function and open new opportunities to therapeutically modulate these pathways.

Beyond the BBB, peripheral inflammatory responses can gain access tothe CNS via the meninges, the multi-layered protective tissue thatsurrounds the brain and spinal cord [reviewed in Rustenhoven and Kipnis (2019)]. Cytokines can accumulate in the dural CSF and cross into the brain, passing between endothelial cells that lack tight junctions (Louveau et al., 2015). Additionally, cytokine signaling specifically within the meninges has been shown to alter neuronal function by binding directly with receptors on neurons in frontal cortical regions and altering cognitive and social behaviors in mice (Derecki et al., 2010; Filiano et al., 2016). Meningeal T-cell production of multiple inflammatory molecules, including IL-17, IL-4, and INF-γ, have been shown to alter both excitatory and inhibitory circuitry and modulate cognitive function and social behavior (Derecki et al., 2010; Filiano et al., 2016; Ribeiro et al., 2019). Lastly, the CNS meningeal lymphatic system also offers a route for peripheral-central immune crosstalk. Since the brain does not contain a resident lymphatic system, waste removal is facilitated by cerebrospinal fluid draining through the meninges into the deep cervical lymph nodes, where interactions between CNS immune molecules and peripheral immune cells can occur (Louveau et al., 2015, 2018). In this manner, the peripheral immune system can gauge central immune status. In the aging brain, dysfunction of the meningeal lymphatic vessels leads to accumulation of harmful amyloid beta-protein toxicity and increase Alzheimer’s pathology (Da Mesquita et al., 2018). Longitudinal imaging studies have shown that progressive brain matter loss is consistent with accelerated aging in patients with SCZ (Schnack et al., 2016). It remains to be determined whether therapeutic agents that boost lymphatic function by either increasing the diameter of the lymphatic vessels or cerebral spinal fluid drainage (Da Mesquita et al., 2018) could improve the cognitive and social deficits observed in SCZ.

## Discussion

### Is SCZ an Inflammatory Disease?

There is growing evidence from both human and animal studies that many of the risk factors for SCZ converge on their ability to promote neuroinflammation, and that these effects are mediated in part by microglia. However, is there a pro-inflammatory phenotype in SCZ? Post-mortem and clinical studies show an increase in pro-inflammatory markers in people with SCZ compared to controls (Fillman et al., 2016; Sekar et al., 2016; Boerrigter et al., 2017; Lesh et al., 2018; Goldsmith and Rapaport, 2020; Pedraz-Petrozzi et al., 2020). Moreover, there is evidence for elevated levels of cytokines in blood samples from people with SCZ, whether they are medication-naive or receiving antipsychotic treatment, during episodes of psychosis (McKernan et al., 2011; De Picker et al., 2019; Mondelli et al., 2020; Steiner et al., 2020). Thus, such studies suggest that inflammation might contribute to the development of SCZ and also drive its progression and cyclic nature.

Schizophrenia cases can be sub-divided using either serum or post-mortem brain tissue levels of pro-inflammatory cytokines, which reveal that about 40% of SCZ cases have a high inflammatory expression signature (Fillman et al., 2016; Boerrigter et al., 2017; Cai et al., 2018). Although these studies suggest there are subtypes of SCZ patients, they do not provide information on their inflammatory states earlier in the disease development nor do they assay inflammation in the brain, which could differ from blood or CSF biomarkers of inflammation. There has been some success in longitudinal PET imaging studies that measure expression of translocator protein (TSPO), a non-specific marker of pro-inflammatory microglia, in the brain (Selvaraj et al., 2018). These studies show that SCZ is characterized by increased TSPO expression, which correlated with greater gray matter loss (Selvaraj et al., 2018). However, there have been mixed results concerning PET measurements of TSPO with some studies showing increased TSPO binding in SCZ (Doorduin et al., 2009; Bloomfield et al., 2016) and others showing no correlation (Di Biase et al., 2017; Notter et al., 2018). Additionally, recent work revealed that neuronal activity can also drive the expression of TSPO (Notter et al., 2020). It is thus not clear if TSPO is a reliable marker for neuroinflammation (Sneeboer et al., 2020). The identification of more specific *in vivo* markers for neuroinflammation would be useful. Ideally, additional work should be done to specifically interrogate the extent of neuroinflammation in SCZ, in addition to peripheral inflammation, to determine if increased inflammation correlates with all or only a percentage of SCZ cases.

Given that SCZ is a highly heterogeneous disease, it is not surprising that there are different disease subtypes. Studies that have divided individuals with SCZ based on inflammatory markers have found more severe symptomology in those with higher levels of pro-inflammatory markers. Specifically, there is evidence for greater gray matter loss and poorer performance in language tasks (Fillman et al., 2016) and increased depressive symptoms (Bossù et al., 2015) in SCZ cases characterized by high inflammatory state. Consistent with this, therapeutics that reduce inflammation provide the greatest symptom improvement in neuropsychiatric cases associated with high inflammation. For example, inhibition of TNF was shown to improve symptoms in people with major depression, but only in those with heightened immune biomarkers (Raison et al., 2013; Weinberger et al., 2015). Additionally, various anti-inflammatory agents including aspirin, estrogen, N-acetylcysteine, COX-2 inhibitors, minocycline and fatty acids (Sommer et al., 2014) have been shown to improve symptom severity in SCZ, but there are some mixed findings of the efficacy of these therapeutics. Minocycline has been shown to reduce microglia and complement-dependent synapse removal in an *in vitro* model from patient-derived neuronal cultures while decreasing the risk for SCZ when administered to young adults (Sellgren et al., 2019), suggesting that targeting synaptic pruning via neuroinflammation would be therapeutic for SCZ and might directly target the disease process. Nevertheless, it is possible that there are discrepancies concerning the ability of some of these drugs to improve symptoms in SCZ because they might only be effective in high-inflammatory cases. Future work aiming to elucidate the differences between subtypes of SCZ could potentially allow for the development of more effective and targeted therapeutics. Although people with SCZ can be divided based on extent of inflammation, there is no denying the role of the immune system in this complex disease.

In line with this, microglia are significantly altered in SCZ and contribute to neural dysfunction by responding and contributing to neuroinflammatory signaling (Figure 3). In SCZ post-mortem tissue, microglia have been noted to have altered morphologies and densities in brain regions known to contribute to the symptomology of SCZ. Microglia engulfment of synaptic material is essential for the normal wiring of the brain and can contribute to pathological states when mis-regulated (Wake et al., 2009; Tremblay et al., 2010; Paolicelli et al., 2011; Schafer et al., 2012; Dejanovic et al., 2018; Filipello et al., 2018; Vainchtein et al., 2018; Weinhard et al., 2018; Comer et al., 2020). There is also evidence that microglia contribute to synapse formation during development, adolescence and into adulthood (Parkhurst et al., 2013; Miyamoto et al., 2016; Akiyoshi et al., 2018; Weinhard et al., 2018). Additionally, a two-photon *in vivo* imaging study in awake mice has shown that microglial contacts with synapses increase synaptic activity thus enhancing neuronal network synchronization (Akiyoshi et al., 2018). In this study, when MIA was induced with poly(I:C), microglia became reactive while neuronal synchronization decreased (Akiyoshi et al., 2018), suggesting that microglia contribute to network function and that their role in this process can be easily disrupted by immune responses.

Although there is evidence that microglia contribute to excessive synaptic pruning in SCZ, it is not clear if microglia-dependent synapse formation is also altered. Since much of the data collected from individuals with SCZ is from post-mortem samples, it is difficult to discern what is occurring on the synaptic level earlier in development. Recent studies suggest that more immature spine types can be differentially targeted in SCZ (MacDonald et al., 2017; Comer et al., 2020), therefore, it is possible that synapse formation mediated by microglia is also altered in SCZ. In a prenatal ventral hippocampus lesion model for SCZ, microglia displayed altered density, morphology and ultrastructure, together with increased expression of multiple complement genes including *C1q* and *C3* (Hui et al., 2019). This increase in microglial expression of complement proteins coincided with an increase of synaptic pruning in the PFC and behavioral deficits in rats, but was reversed by administration of minocycline (Hui et al., 2019). These studies highlight the necessary role exerted by microglia in normal brain development but also show their ability to drive neuroinflammation and contribute to pathology in disease states.

Indeed, there are multiple disease-associated microglial subtypes such as those seen in neurodegenerative disorders (Deczkowska et al., 2018) and dark microglia which were recently observed in SCZ post-mortem brain samples (Uranova et al., 2018). More work is needed to fully understand microglial subtypes that are more prevalent in disease states and how they contribute together to pathology, however data suggest they partially contribute to disease by enhancing synaptic pruning (Stratoulias et al., 2019). Future studies should also aim to develop more translational animal models so that *in vivo* studies can be performed to gain greater understanding into how microglia functionally impact synaptic development and circuit function in pathological states.

### Does Inflammation Affect Specific Circuits and Neuromodulatory Systems?

Although there is no doubt that the immune system plays a critical role in shaping brain development and contributes to disease states when dysregulated, there is a need to understand which specific circuits and neuromodulatory systems in particular are most impacted by abnormal immune signaling. It is clear that complement proteins facilitate the removal of synapses (Stevens et al., 2007) and that the upregulation of complement proteins contributes to circuit miswiring (Comer et al., 2020). However, SCZ is also characterized by alterations in inhibitory circuits (Dienel and Lewis, 2019), neuromodulatory systems such as dopamine (Howes et al., 2017) and glutamate (Uno and Coyle, 2019), and changes in the connectivity between brain regions such as the hippocampus and PFC (Sigurdsson and Duvarci, 2015). Do inflammatory responses alter specific neurotransmitter systems and networks differentially?

There is evidence that inflammatory responses target specific neuromodulatory systems and brain circuits. For example, changes to the gut microbiome driven by inflammation can alter the production of serotonin (Rogers et al., 2016), which is known to be disrupted in SCZ. Interestingly, MIA in rats has been found to increase the levels of dopamine in both the nucleus accumbens and mPFC (Luchicchi et al., 2016) of offspring, which is well-known to play a role in the positive symptoms of SCZ (Kesby et al., 2018). Additionally, MIA initially triggers hyperinhibition and neuronal miswiring, before leading to a reduced inhibitory drive (Thion et al., 2019). ELS in mice was shown to alter HPA circuity development, in addition to hippocampal and PFC function (Brenhouse et al., 2019). In addition, ELS is known to have an impact on inhibitory connectivity (Goodwill et al., 2018; Ohta et al., 2020), and previous work suggests that oxidative stress and/or neuroinflammation might underlie the changes in parvalbumin interneurons in response to ELS (Holland et al., 2014; Brenhouse et al., 2019). There is evidence for parvalbumin interneuron dysfunction in SCZ, as they have altered density in the frontal cortex of individuals with SCZ (Kaar et al., 2019). Interestingly, the meninges modulate cortical interneuron migration during development (Borrell and Marín, 2006); future work could interrogate whether changes in meningeal signaling, such as immune molecule signaling, could contribute to alterations in interneuron migration in SCZ.

**FIGURE 4:**
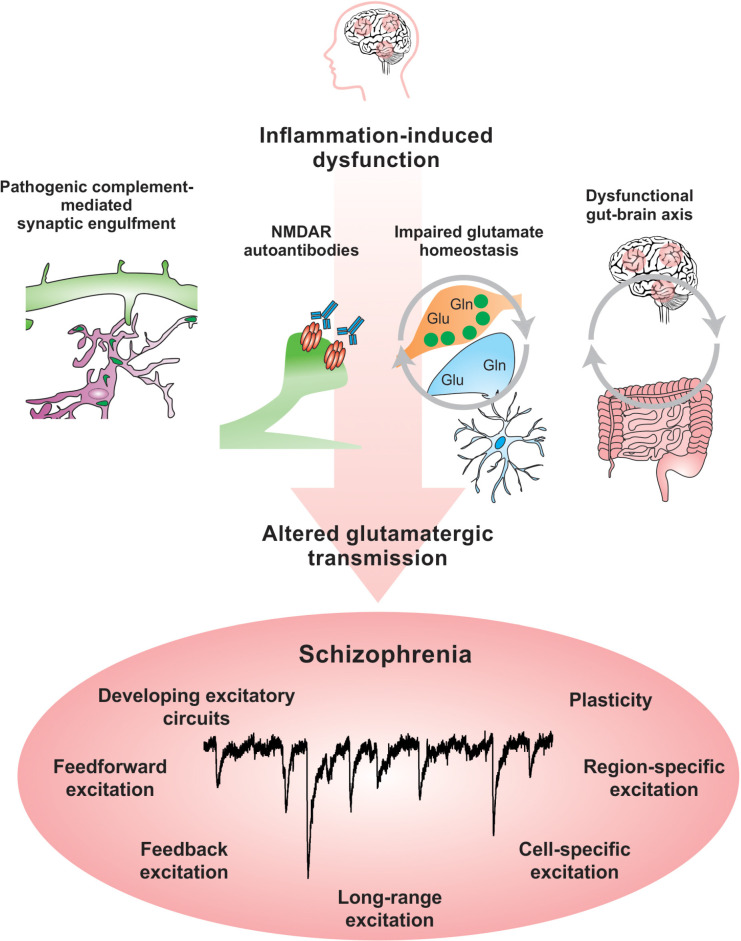
Neuroinflammation-induced dysfunctions that alter glutamatergic transmission in SCZ. Changes in glutamatergic transmission are known to occur in SCZ. Neuroinflammation impacts excitatory circuitry in SCZ through complement-mediated engulfment of excitatory synapses, the production of autoantibodies against NMDARs, changes in glutamate homeostasis potentially mediated by alterations in astrocytes and changes in the gut-brain axis, a known regulator of glutamate synthesis that is able to impact CNS functions such as stress responses. These changes in excitatory transmission can alter brain circuitry, for example, by altering synaptic plasticity and long-range excitation.

The glutamate hypothesis of SCZ evolved from observations that NMDA receptor antagonists, such as ketamine, produce behavioral states similar to SCZ negative and positive symptoms in healthy human subjects (Krystal et al., 1994; Adler et al., 1998; Hu et al., 2015). As discussed throughout this review, studies have also reported spine dysgenesis and alterations in mRNA and protein levels of glutamate receptors in human SCZ post mortem tissue. The presence of circulating autoantibodies against glutamate and NMDARs in a subpopulation of psychotic SCZ patients further support this hypothesis (Ehrenreich, 2018). It has recently been shown that increased expression of the SCZ-associated gene *C4* led to a decrease in excitatory connectivity with no loss of inhibitory transmission, suggesting that excitatory synapses might be more vulnerable to elimination (Comer et al., 2020). In support of this, neuronal pentraxins, regulators of AMPA receptor trafficking, are interacting partners of C1q (Ma and Garred, 2018), providing a link between the complement pathway and excitatory synapse elimination. Another link between alterations in glutamatergic transmission and SCZ comes from studies in astrocytes, which have increased reactivity in SCZ (post mortem tissue) and are positioned to alter glutamatergic transmission through the regulation of glutamate biosynthesis, release, uptake and metabolism [reviewed in Mei et al. (2018)]. Lastly, the mobile genetic element, human endogenous retrovirus is associated with neuropsychiatric conditions and produces a protein that alters glutamate synapse structure and plasticity dependent on the presence of glial cells and neuroinflammatory signaling, contributing to altered behavior when expressed in mice (Johansson et al., 2020). Together, these studies suggest that inflammation might contribute to altered glutamatergic transmission through multiple mechanisms (Figure 4).

Although these studies suggest that risk factors for SCZ can exert specific effects on different CNS circuits, more work is needed to fully understand the mechanisms by which inflammation alters specific neuromodulatory systems and circuits. Studies that combine mouse models of SCZ and inflammation with whole-brain or mesoscopic imaging could shed light into how specific neuronal networks are impaired in SCZ (Sofroniew et al., 2016; Boido et al., 2019; Grandjean et al., 2020).

### Impact of the Immune System on Synapse Development in SCZ

It is well-established that spine dysfunction is present in SCZ and is at least partially mediated by reductions in excitatory synaptic connectivity and plasticity (Glantz and Lewis, 2000; Glausier and Lewis, 2013; Berdenis van Berlekom et al., 2020). However, most of the work in this field has relied on human post-mortem tissue, so there is limited knowledge of what could be occurring earlier in development to drive these synaptic alterations. Recent work has highlighted the fact that the immune system works closely with the CNS to establish and refine neural circuits in healthy states as a part of normal development (Paolicelli et al., 2011; Tay et al., 2017b; Hammond et al., 2018). However, when this process is dysregulated, it can lead to pathology and the miswiring of the brain through synaptic loss (Schafer et al., 2012; Comer et al., 2020), which occurs in SCZ.

How are certain synapses selectively removed while others are protected? In the developing brain, there is a period of enhanced synaptogenesis followed by critical developmental periods characterized by experience-dependent refinement of synapses (Trachtenberg et al., 2002; Holtmaat and Svoboda, 2009). Synaptic elimination driven by sensory experience refines brain circuitry by optimizing connections between neurons. In SCZ, this process is thought to be dysregulated, thus leading to a loss of both excessive and necessary synapses, causing aberrant brain connectivity. Recent data suggest that more immature spine types, such as filopodia and thin spines (Cruz-Martín et al., 2012), are lost while larger, more established spines remain intact in SCZ (MacDonald et al., 2017). A similar phenotype was seen in mice overexpressing the mouse homologue of the SCZ-associated gene *C4*. In this *in vivo* model, synaptic loss observed in the PFC was specifically due to a loss of smaller spine-types while mushrooms spines were unaffected (Comer et al., 2020). This evidence is in line with previous work showing that complement-dependent synapse removal is activity-dependent and that connections with less activity are more likely to be eliminated (Schafer et al., 2012). Small spines were also shown to be preferentially contacted and eliminated upon microglial contact *in vivo* (Tremblay et al., 2010). Additionally, it has been suggested that immune signaling is able to protect more mature spines. There is increased expression of the “don’t eat me” signal CD47 at synaptic inputs that are more active (Lehrman et al., 2018). In this way, the immune system would guide synaptic wiring by tagging synapses for removal while protecting other connections that are essential to the function of a circuit. However, more work is needed to support this idea and understand the mechanisms by which the immune system contributes to synapse-specific elimination versus stabilization.

It is also possible that in SCZ, spine loss is driven by the inability of circuits to produce “appropriate” connections. Therefore, the subsequent excessive pruning that occurs in SCZ could be due to the fact that neurons fail to produce adequate connections in the first place. This is relevant in the context of microglia since they regulate synaptogenesis (Miyamoto et al., 2016). Since most of the data obtained from people with SCZ is limited to post-mortem tissue, our information about what is happening on the circuit and synaptic level during development is limited. In this scenario, using mouse models to understand the role of microglia and immune signaling in synapse formation during the first weeks of postnatal development, when most synaptogenesis occurs (Cruz-Martín et al., 2010), is key. Future advances in the resolution and capabilities of *in vivo* human imaging studies notably through specific markers could help answer this question. It is encouraging that previous studies show a similar phenotype in mice that is seen in humans in terms of weaker synapses preferentially being eliminated (MacDonald et al., 2017; Comer et al., 2020). This could allow for studies in mice that more readily translate to humans. Understanding the mechanisms of complement-mediated circuit wiring is a worthwhile area of future study given it is both a mechanism of normal brain development and is implicated in multiple neurodevelopmental and neurodegenerative diseases. Lastly, enthusiasm has grown over the last decade to study marmosets in neuroscience research and an increase in the feasibility of genetic manipulations could provide an additional model to study how abnormal neuroimmune signaling contributes to SCZ (Okano et al., 2016; Servick, 2018).

### Risk Factors Likely Interact Synergistically to Increase Odds for Developing SCZ

Here, we have highlighted the diverse risk factors for SCZ and how they impact the CNS by altering immune signaling. Likely, these risk factors act additively on certain signaling pathways to push vulnerable individuals past a certain threshold into a disease state. This field would benefit from future studies that aim to elucidate how the immune system regulates specific circuits and neuromodulatory systems to drive the diverse phenotypes observed in SCZ. Additionally, an in-depth understanding of the specific signaling networks compromised in SCZ may enable the restoration of typical immune-driven neurodevelopment after exposure to the various genetic and environmental risk factors described in this review.

## Author Contributions

AC, MC, M-ÈT, and AC-M wrote the manuscript. All authors contributed to the article and approved the submitted version.

## Conflict of Interest

The authors declare that the research was conducted in the absence of any commercial or financial relationships that could be construed as a potential conflict of interest.
